# Exploring the multidimensional relationships between educational situation perception, teacher support, online learning engagement, and academic self-efficacy in technology-based language learning

**DOI:** 10.3389/fpsyg.2022.1000069

**Published:** 2022-11-17

**Authors:** Xiaoquan Pan

**Affiliations:** Xingzhi College, Zhejiang Normal University, Jinhua, China

**Keywords:** educational situation perception, teacher support, online learning engagement, technology-based language learning, academic self-efficacy

## Abstract

The study explored the multidimensional relationships between educational situation perception, teacher support, online learning engagement and academic self-efficacy in technology-based language learning in a sample of Chinese undergraduate students, and meanwhile examined the mediating effects of academic self-efficacy and teacher support. A total of 392 (126 male and 266 female) Chinese university students reported on their perceived educational situation, teacher support, online learning engagement, and academic self-efficacy. Results showed that educational situation perception was significantly and positively associated with teacher support, online learning engagement and academic self-efficacy; teacher support and academic self-efficacy was positively correlated with online learning engagement. More importantly, academic self-efficacy as well as teacher support mediated the relationship between educational situation perception and online learning engagement. These findings extended previous research by considering both the external factors (i.e., educational situation; teacher support) and the internal factors (i.e., academic self-efficacy) of influencing students’ online learning engagement in technology-based language learning, thereby contributing to enhancing our understanding of the joint drive of the inherent and extrinsic power mechanisms. This study highlighted the following aspects: (1) strengthening the consideration of the key elements of the educational situation; (2) clarifying the pivotal position of intelligent technology in educational situations; and (3) emphasizing the reconstruction of intelligence teaching ecology driven by learning activities. Besides, this study indicated the significance of elevating teachers’ awareness, willingness and capacity of the substantial supports in enhancing students’ online learning engagement and would inform that the future research on the connotation and ways of teacher support should be responding to technology-based learning environments.

## Introduction

With the rapid development of emerging intelligence technology such as big data and artificial intelligence, the intelligent technology as an endogenous variable has been gradually embedded in teaching (Chau et al., 2021). As can be predicted, the future pedagogical ecology will gradually present a developmental trend of intelligence-based situation perceptions and technology-enhanced collaborations, which not only helps teachers leverage technologies to adapt instructional strategies to students’ individualized learning needs (Lawless and Pellegrino, 2007), but also elevates the complexity of the classroom teaching contexts. Thus, how to use the advanced educational theory and intelligent technology to achieve the ecological construction of teaching matrix, and how to clarify the occurrence mechanism of classroom teaching under the background of emerging intelligent technology, has become a pivotal problem that has increasingly been embedded within a social and technological framework (Nevgi et al., 2006; Goodyear et al., 2014; McKenney and Reeves, 2018). For this reason, the related research on the educational situation perception, which attempts to capitalize on the various visible and invisible routes to work out good technical solutions, enhances our understanding of this issue by identifying the influence of technology-based environments on teaching and learning, such as using intelligent perception technology to realize the analyses of the entity elements in intelligent learning space (Olsen et al., 2020), the deconstruction of classroom teaching ecosystem (Castañeda and Selwyn, 2018), the customization of technology-based learning supports (McLoughlin and Lee, 2010; Glover et al., 2016), and the reconstruction of classroom teaching ecology in relation to intelligent technology (Bower and Vlachopoulos, 2018).

One of the fundamental goals of scientific research in language learning is to identify the conditions or factors that drive learners’ language acquisition (Yang, 2018). Arising from these efforts, several theories, and hypotheses have been constructed as frameworks to enable researchers to examine significant variables that can be used to predict and explain language acquisition mechanisms at individual and organizational facets. For instance, the existing literature has a lot to offer in terms of the influence mechanism of language learners’ knowledge skills, cognitive level, learning motivation, learning attitude, and other potential characteristics on the language learning process and learning outcomes, so as to explore the internal mechanism of the occurrence of language learning (Inozu et al., 2010; Benson and Reinders, 2011). There have also been studies focusing on the external educational situations related to language learners, so as to explore how teacher support, teaching resources, learning environments and other educational situation factors exert effects on students’ learning engagement, and reveal the influence mechanism of educational situation factors on students’ learning (Levy, 2009; Reinders and White, 2011). However, the current literature has a spatial of being extended to conduct accurate analyses on the external educational situation related to language learners, to explore how educational situation elements (e.g., teacher support, teaching resources, and learning environments) affect the learners’ inner knowledge construction, cognitive development and emotional state, and to reveal the influence mechanism of educational situation factors on the occurrence of language learning. The current study intends to provide some insights into the issue and thus helps enhance our understanding of the influence of educational situation perception on students’ language acquisitions.

Technology with its fast-moving pace has pervaded the educational aspects in recent years (Garrison and Akyol, 2009; Hung et al., 2010), thus enabling students’ self-initiated, self-constructed, and self-monitored learning experiences in a newly-constructed technology-based ecology of language learning (Lai and Gu, 2011; Reinders and White, 2011). Combing the current research, we found that the related research concerning technology-based learning primarily focuses on the utilization of artificial intelligence technology to analyze and expound learners’ learning behavior, cognition, and emotional state perceptions, or mainly adopts empirical research methods to explore the influence mechanism of the educational situation elements on learners’ academic performance and their emotional state. For instance, exploiting this theory and analysis method, the field of pedagogical research sees the verification and explanation of the purpose of technology use (Teo and Noyes, 2014), the structure equation analysis of the factors influencing students’ network learning (Teo, 2010), the research on the technology use in self-directed language learning beyond the classroom (Lai, 2015; Lai et al., 2016), and the characteristics of students’ technology use for extracurricular language learning (Lai et al., 2018), etc. These studies are the specific applications of intelligent technology in the empirical contexts, but these studies could be further extended by integrating intelligent technology into individual practical problems so as to form a sophisticated understanding of the educational potentials of technological resources, of the variety of technological resources students could utilize and of how to use technological resources effectively for learning (Kennedy and Miceli, 2010; Lai, 2015). For example, in addition to the educational situation constructed by the network ecological environments, students’ academic self-efficacy, learning engagement, and other subjective factors, as well as the external factors of teacher support, will also affect students’ technology-based language learning. Based on this, this study intends to explore the relationships between educational situation perception, teacher support, students’ online learning engagement and academic self-efficacy, aiming to deeply integrate the related researchers on “situational perception,” “teacher intervention,” and “learners’ behavioral engagement,” and to explore the influence mechanism of external educational situation elements (educational situation and teacher support) on learners’ learning engagement by reconciling the frontier technology of educational data mining (Cukurova et al., 2019). Besides, by analyzing the learners’ academic self-efficacy, this study attempts to explore the influence of students’ internal cognition and emotional development, so as to reveal the deep occurrence mechanism of learning behaviors.

## Literature review

### Educational situation perception

In recent years, the introduction of emerging intelligent technologies such as the networking, big data and artificial intelligence has brought tremendous changes to the lives of the whole people, which is reflected in higher education in the educational ecological environment formed by the integration of the new generation of information technology. The educational situation constructed by the emerging technology not only effectively supports language teaching, but also changes the educational ecology of language teaching. Confronted with the strong penetration of information technology, language learners must adapt to this educational situation. The so-called “situation” usually means any information that can be used to identify entity states like people, objects, environments, and computer programs (Dey, 2001), which is primarily to characterize the existence morphology and evolution patterns of various entity elements under specific spatial and temporal conditions. The concept of situation perception first derives from universal computing which is to acquire the situational parameters in the environment through sensors and related computing devices, obtain useful feedback information to users through machine processing, and realize the interaction and fusion between users and the environment with the help of computing devices.

On the theoretical level, the conception of situation perception holds that knowledge is an interactive state constructed in the process of the interaction between individuals and the environment, contends that learning is the construction of individual meaning completed in authentic situations and practical activities, and meanwhile emphasizes the influence mechanism of the creation of educational situations on the cognitive development of learners (Chu et al., 2010; Healey et al., 2010; Hwang and Chang, 2011). In recent years, in the field of learning science, the study of the influence mechanism of media, resources, environments, learning behaviors and other factors on learners’ cognitive and emotional development also confirmed the complex functional relationship between “situation” and the learning occurrence mechanism of learners (Hung et al., 2013; Martin and Ertzberger, 2013; He and Li, 2019; Cui et al., 2022). This category of study accords with the notion of *learning ecology* put forward by Barron (2004), who defined as “the accessed set of contexts, comprised of configurations of activities, material resources and relationships, found in co-located physical or virtual spaces that provide opportunities for learning” (p. 6). Based on this, the research on the occurrence mechanism of learning in the intelligent era should pay much more attention to the learners’ internal characteristics such as prior knowledge, intellectual level, and emotional motivation, as well as the complex effect mechanisms of the external environmental factors such as teaching content, teaching media and teaching activities on the learning process and academic outcomes.

At the technical level, the rapid development of intelligent technology provides the underlying technical support for the development of learners’ modeling and educational situation perception research (Papachristos et al., 2013; Dessi et al., 2019), which is specifically manifested in the following aspects. First, various intelligent perception devices are used to realize the intelligent data collection of learners, teachers, teaching resources, environments and teaching activities, and build a multimodal data set for learners and educational situations. Second, technologies such as language processing, computer vision, speech recognition, and physiological information recognition are utilized to attain the effective mining of learners and educational situation characteristics, and to effectively restore the representational forms of things from multiple levels. Third, the method of data mining is adopted to achieve the mining and analysis of the complex correlation between learners and the educational situation, and explore the influence mechanism of the creation of the educational situation on the development of learners’ potential characteristics, so as to promote the in-depth development of relevant research.

The learning behavior research which builds on situation perception emphasizes the influence mechanism of the external educational situation elements on the learners’ cognitive and emotional state, and explores the interaction mechanism between teaching activities and the educational situations (Mavrikis, 2010; Kulik and Fletcher, 2016). In addition, the learning behavior research tries to establish a two-way matching mechanism between educational situation characteristics and learner behaviors, and attempts to explore the association between educational situation elements and learners’ behavior characteristics by analyzing the educational situation and learners’ behavior under multiple learning space and time conditions (Cheng et al., 2016; Waheed et al., 2020). The main difference between the learning behavior research based on situational perception and the traditional learning behavior research is that the traditional learning behavior research pays more attention to the analyses of potential characteristics such as learners’ knowledge structure, cognitive behavior, emotional state, learning preference, and learning motivation, whose goal is to accurately depict the cognitive structure of learners so as to provide personalized learning support services. While the learning behavior research based on situational perception emphasizes more the effect of the educational situation elements on the learners’ inner cognitive and emotional state (Liyanawatta et al., 2021), and constructs the relationship between education situation factors and learners’ characteristics through the data analysis of learning behaviors and education situations (Gu et al., 2019). Fundamentally speaking, the study of learning behavior based on situational perception focuses on the exploration of “educational situation,” “learners,” and “the interactive relationship between learners and educational situation,” aiming to conduct a three-dimensional and comprehensive analysis of the learning behavior process from a broader level (Malmberg et al., 2019).

With the integration and development of modern learning theory and the network technology environment, relevant researchers pay more and more attention to the influence mechanism of educational situation creation on the occurrence of teaching activities and students’ cognitive development, and have made a series of attempts (Miao et al., 2006; Plass et al., 2015; Thorburn and Stolz, 2021). For instance, Yeoman and Wilson (2019) designed situated learning, involving the learner situation and the teaching service situation, and based on this, a ubiquitous learning-oriented learning resource retrieval model was constructed to realize the deep aggregation and dynamic push of teaching resources through the accurate perception of the educational situation. Similarly, by highlighting the support of educational technology, the study of Sharan (2015) was deployed to create a situational learning environment to encourage learners’ participation in a meaningful learning process that allows them to construct knowledge through their experiences, feelings, and collaboration. This was alignment with Gawande and Al-Senaidi (2015) who explored situated learning and constructed a learning resource recommendation system based on situational perception, which divided educational situation elements into learning objectives, learner characteristics, learning facilities, and learning environments. Through accurate perception and integration analysis of educational situation in ubiquitous learning environments, precise, timely and actionable information was provided, helping students learn in a meaningful, relevant context (Plass et al., 2015). Following on from this, Lu et al. (2022) investigated the relationship among situational engagement, personal characteristics and learning environment perceptions oriented to intelligent learning environments, aiming to realize the dynamic optimization of intelligent learning space by using situational perception technology. On the whole, as education situation perception is the hot topic of the field of intelligent education, some applications of these models/theories are found in underpinning the studies examining the construction of the intelligent learning environment (Aleven et al., 2017), the adaptive learning support service based on situational perception (Bligh and Crook, 2017) and the modeling of learners’ learning behaviors based on situational perception (De Corte, 2012). These studies have profound implications for the development of intelligent education. However, in terms of the essence of learning, in addition to the influence of external factors such as teaching environment, learner behavior is also influenced by internal factors such as prior knowledge, intellectual level, learning ability, and learning attitude. In addition, language learners also differ in information technology literacy and self-directed learning engagement, so teachers’ teaching intervention (e.g., teacher support) plays a critical role.

### Impact of teacher support on online learning engagement

Teacher support arises from the students’ learning process, which is the supportive behavior obtained by students in their study (Hughes et al., 2008; Roorda et al., 2011). Teachers significantly shape the quality of students’ learning experiences by affecting students’ cognitive, affective and social learning behaviors (Farmer et al., 2011). As a significant social agent, teachers play a critical role in helping students develop autonomy of technology-based language learning beyond the classroom (Reinders and Darasawang, 2012). “In light of these particular research lines, the function of teacher supports should be manifested in helping students to be academically, professionally and psychologically empowered, motivating students’ personal attribute, and facilitating students’ self-initiated use of technological resources to autonomously clutch the reins of self-directed learning process” (Pan and Chen, 2021, p. 3). Despite of different characteristics and functions of teacher supports, researchers have classified teacher support into three categories: (1) teacher affective supports, mainly referring to teacher behaviors which can provide students with the basic knowledge of the strengths of technology as well as the encouragement of using technology in language learning (Xia and Lee, 2000); (2) teacher behavior supports, involving teachers’ capacities of organizations and management that can help students participate in activities and tasks involving technologies (Ertmer, 2005); and (3) teacher capacity supports, mainly helping students to get some useful technological resources and tell them how to select and use technological resources effectively (Gallivan et al., 2005). According to social support theory, support behaviors acquired or perceived by individuals from social relationship networks are generally beneficial and promote individual mental health and development (Hughes et al., 2008). As one of the social support system, teacher support exerts a certain impact on students’ academic performance. Additionally, teacher support, as an external environmental factor of accelerating students’ positive development, was examined to significantly predict learning engagement (Rubin et al., 2015).

The role of teachers is crucial in the online teaching and learning process. Therefore, examining the constituent dimensions of teachers’ support strategies and clarifying their influence on students’ online learning engagement plays a positive role in effectively improving the effectiveness of online teaching. In the context of technology-based learning environments, teacher support constitutes an important influence factor that can determine students’ adoption and utilization of technologies for online learning (Lai and Zheng, 2018; García Botero et al., 2019). Previous studies have explained teacher support functions, such as teacher support into providing emotional encouragement, alternative choices, positive feedback, exchanging views, and allowing students to work in their own way (Hughes and Chen, 2011). Similarly, Lam et al. (2012) explored the positive influence relationship of learners’ perceived teacher support on their learning motivation and academic performance in the bilingual teaching situation.

Learning engagement, as a key factor in the learning process, plays a positive role in improving academic performance. Many researchers have found that the higher the perceived level of teacher support is, the more time and energy students invest in learning (e.g., Carson and Mynard, 2012; Hew, 2016; Jung and Lee, 2018). Kearsley and Shneiderman (1998) proposed engagement theory as a model of technology-based environment learning, and stated that learning engagement can be accomplished through an emphasis on collaborative efforts, project-based assignments, and a nonacademic focus. O’Brien and Toms (2008) sought to critically deconstruct students’ engagement experiences with technology and found a common trajectory, as their technology-based learning engagement partly resulted from collaborative efforts. From the perspective of ecosystem theory, learning engagement is closely related to its learning environment (Coll et al., 2014). The development of network technology has made online learning a new learning paradigm. Online learning engagement refers to the degree of student behavioral, cognitive and emotional participation in the online learning process with the help and guidance of teachers. Students’ physical and mental engagement in online learning significantly affects their academic performance (Fang and Zhang, 2012), so how to improve students’ participation in online learning has always been one of the important issues in the field of education. In the field of second language teaching, Lai (2015) examined the teacher support in the online teaching environment and its interaction with language learners’ learning behaviors, showing that online teacher support mainly consists of affective support, behavioral support, and capacity support, and teacher support has a positive impact on students’ interactive learning engagement. Despite researchers have confirmed the critical role of teacher support for students’ learning, empirical studies which explored the impact of teacher support on students’ learning engagement in online situations are insufficient. Based on this, the analysis of the different effects of students’ perception of teacher support on English learning engagement during online learning is the main focus of this study.

### Academic self-efficacy as a mediator between educational situation perception, teacher support, and online learning engagement

According to reciprocal determinism put forward by Bandura (2001), there are relatively independent and causal relationships between individual behaviors, person factors and external environments. Among them, the person factor is characterized as the human physiological response capability, cognitive ability and other physical and mental functions. The reciprocal determinism transcends the “one-dimensional determinism” of traditional cognitive psychology, taking the individual, environment and behavior into account and building a bridge between an individual’s internal cognition and external environment around behavior. In the technology-based educational situation, teacher support plays an irreplaceable leading role. On the one hand, learners have a clear learning task in online learning, whose individual behaviors entail teachers’ supervision, management, and feedback. On the other hand, the person factors of students are easily influenced by teachers, producing two completely different learning behaviors: active participation or negative participation. In terms of students’ main psychological factors, their academic self-efficacy constitutes a key component. Academic self-efficacy derives from the classic psychological concept of self-efficacy, which refers to the students’ confidence and ability to identify whether they can complete a certain task (Schunk, 1991). According to Yesilyurt et al. (2016), self-efficacy consists of the regulation of cognitive, social, emotional, and behavioral skills required in order to perform a task and applying effectively to the situation. Academic self-efficacy is reflected in an individual’s confidence in their ability to successfully complete academic tasks at a specified level. Based on Bandura’s social cognitive theory and the concept of efficacy, scholars have developed many tools for measuring academic self-efficacy, mainly classified into two main categories. The first category is used to measure an individual’s confidence in their ability to perform course-specific tasks, such as the Usher and Pajares (2009)‘s mathematical self-efficacy scale. The second category is the self-efficacy scale suitable for more general academic behaviors, such as the one-dimensional academic self-efficacy scale of Yilmaz et al. (2007). Although the measures were slightly different, they were all developed based on the social cognitive theory and Bandura’s concept of self-efficacy, and thus their connotation was basically alike. Relevant research literature suggests that academic self-efficacy may be an important intermediary variable of student’s perception of teacher support affecting students’ learning engagement for the following two reasons. First, student-perceived teacher support is an important factor that cannot be ignored in the formation of students’ academic self-efficacy. According to the theory of self-efficacy (Bandura, 1997), individuals who feel the respect and encouragement of others can improve their self-efficacy, and respect and encouragement are the specific manifestations of teachers’ support behavior. Some empirical studies also found that all perceived support had positive predictive effects on academic self-efficacy (Affuso et al., 2017; Xu and Qi, 2019). In other words, students who perceive a higher level of teacher support have a higher level of academic self-efficacy. Secondly, academic self-efficacy can affect students’ learning engagement, as self-efficacy is the result of an individual’s ability, which can affect their choices and the result of specific behavior.

Research shows that teacher support can significantly affect students’ academic self-efficacy and play a positive role in their learning confidence and academic ability (Affuso et al., 2017). Positive feedback such as praise and reward can enhance students’ academic self-efficacy, while negative feedback such as criticism and punishment will weaken their academic self-efficacy. In a more caring, challenging, skill-oriented learning environment, and students’ self-efficacy in learning was significantly higher. In short, effective teaching support has a strong impact on academic self-efficacy from multiple aspects. This effect was further reflected in a significant impact of teachers’ effective teaching support on students’ classroom participation (Alivernini and Lusidi, 2011). In teaching, teachers’ incentives and recognition constitute a significant impact on students’ behavior, cognition, emotional engagement, and classroom participation, and can stimulate students to participate in classroom frequency and depth. Meanwhile, students’ academic self-efficacy is closely related to their classroom participation. Studies have found that one of the main reasons for students’ low classroom participation is their lack of confidence in learning (Klem and Connell, 2004). These subjective feelings of the students are the relatively low explicit manifestations of their academic self-efficacy. In addition, in the process of online learning, students’ enthusiasm for independent learning engagement is not high enough, which is caused by the influence of academic self-efficacy factors. Research shows that academic self-efficacy can significantly affect students’ online learning engagement (Bassi et al., 2007). For example, students with high academic self-efficacy will think and discuss more actively to meet the challenge of learning; otherwise, students with low academic self-efficacy will tend to participate negatively or even escape from online learning engagement.

According to the teacher expectation model, teachers’ high expectations to students affect teachers’ instructional behaviors, and then students produce internal psychological changes through their perception of teachers’ behavior, ultimately influencing their learning engagement (Klem and Connell, 2004). Behaviors such as learning support and emotional support offered by teachers are proved to be important factors affecting students’ learning engagement. According to Bandura (1997)‘s point of view, self-efficacy belief is the product of self -persuasion process, which depends on the active, social, and physiological functional information of cognitive processing and functional faith, and thus self-efficacy will greatly promote individual function level and quality. Teachers’ trust of students and positive evaluation/feedback can effectively improve students’ academic self-efficacy. In addition, previous research found a significant positive impact of teachers’ affection, capacity, and behavior support on their academic self-efficacy (Lai, 2015). Longitudinal studies and meta-analytical literature supported that students with higher academic levels of self-efficacy demonstrated higher academic goal setting, greater emphasis on academic performance, more time spent in learning engagement, and higher academic performance (Hughes and Chen, 2011; Roorda et al., 2011).

In recent years, researchers have expanded the research on teacher support based on the vision of intelligent education. Developing a student-centered and intelligent teacher support service system has become an important way to explore intelligent education. In the environment of intelligent education, more concerns should be aroused on learners’ perception of teacher support, and more attention should be paid to teachers’ knowledge guidance, tool navigation, social support and emotional support. Modern network technology imparts infinite possibilities for better integrating the educational situation, improves the learning experience of students, and enhances their perception of the real learning situation. In this context, scholars have actively explored and tried the theory and practice of intelligent education, involving the analysis of the system structure and key technologies of intelligent education from the technical point of view, the interpretation of the practical situation and existing problems, and the discussion of the realization path of intelligent education (Kulik and Fletcher, 2016; Blau et al., 2019). However, it can be found that these studies all weaken the supporting role of teachers as the “core manpower” of the intelligent education environment. In fact, the integration of technology into education is by no means the substitution of technology for teachers, but rather enriches and expands the connotation and function of teachers. In the context of technology use for online language learning, students’ perception of teacher support can enhance students’ self-efficacy, motivate students’ behavioral, cognitive and emotional engagement, stimulate learning motivation, and improve learning adaptability and academic performance (Ertmer, 2005; Reinders and Darasawang, 2012; Lai et al., 2016). From these literatures, teacher behavior, attitude and expectation, teaching methods, pedagogical task design, and learning feedback will affect online learners’ online learning engagement.

In short, under the condition of network technology, the composition of network ecological environment is divided into two parts: “situational environment constructed by technology” and “subject community.” Technology-constructed educational situation is the network ecological environment for learners to develop technology-based language learning; while the “subject community” embodies the behavioral characteristics of the individuals’ acting in the network environment. Self-determination theory put forward by Deci and Ryan (2000) concentrates largely on how the environments affect people’s basic psychological needs for autonomy, competence, and relatedness (Jeno et al., 2019), which can be used to comprehend the enhancement of learners’ online learning engagement through the effects exerted by the educational environments (Chiu, 2021). Besides, responding to the network ecological environment, scholars have conducted the ecological exploration of language teaching, such as focusing on the discourse analysis of teacher-student interaction and peer interaction (Gawande and Al-Senaidi, 2015), the online interactive network structure characteristics of adult learners in the network environment (Sabah, 2016), and the construction and implementation path of personalized intelligent teaching model (Cui et al., 2022), and so on. However, the existing studies rarely discuss the influence of teacher support and educational situation on the online learning engagement and its influence mechanisms. In particular, few studies combined students’ academic self-efficacy with online learning engagement to further explore the mediating role of learners’ internal psychological mechanisms.

### Research questions

Informed by the above discussed new visions in technology use for educational research, the overarching research questions for the present study are as follows:

1. What are the contributions of educational situation perception, teacher support, and academic self-efficacy to students’ online learning engagement in technology-based language learning?

2. Will academic self-efficacy and teacher support mediate these relationships?

## Methodology

### Participants and procedure

The participants of this study are sophomores of college English course from the university where the author works. In response to the utilization of technology in teaching, the college English teaching and research group has actively involved in the initiatives of teaching innovations and adopted a blended teaching method. In addition to normal classroom-based teaching, college English teaching and learning was conducted through a unified network platform, “where teachers and students interact, and teachers give feedback and evaluate students’ learning” (Pan and Shao, 2020, p. 3). Besides, the relatively uniformed standards in teaching guidance and target requirements were formulated. As for the choosing of sophomores as research participants, two main factors are considered: (1) college English, as a compulsive foreign language for non-English majors, has a total of two academic years in Chinese universities; (2) currently, modern network technology is widely used in college English teaching. Through one academic year of technology-based college English learning experience, students have their own understanding of educational situation perception, teacher support, online learning engagement, and academic self-efficacy, which facilitates the development of this research.

This study randomly selected several parallel classes, through on-site face-to-face distribution of paper questionnaires at intervals before class. The questionnaire survey lasted about 10 min, and recycled immediately. In this study, 410 questionnaires were distributed and 398 were collected, with a collecting rate of 97.1%. Among the collected questionnaires, 6 students had missing values when filling in personal background information and answering related survey questions, so their data were deleted during data analysis. Only data from 392 (126 males, accounting for 32.1%) students who responded to the complete items of the questionnaire were analyzed. Before conducting the questionnaire survey, the study obtained the students’ consent and informed them that all the research data collected were anonymized to protect participants’ privacy. Students’ participation was cooperative and voluntary, and thus they carefully completed the questionnaire.

### Measures

#### Educational situation perception

On the basis of the research conducted by Richardson (2006) and Law and Meyer (2011) on students’ perception of classroom environment and the application of network technology in college English teaching, the present study adapted and designed the Educational Situation Perception questionnaire. There are 14 items in the questionnaire, including 4 dimensions: teaching and learning behavior (4 items), teaching resources and service (3 items), physical environment and social environment (3 items) and learning interaction and evaluation (4 items). A sample item is “I knew how to use technology on my own.” A six-point Likert scale was used for the questionnaire items, ranging from 1 (strongly disagree) to 6 (strongly agree). Higher scores indicated higher perceptions of educational situation. The standardized factor loadings (SFLs) of the 14 items range from 0.825 to 0.882, and the Cronbachαvalue and the Kaiser-Meyer-Olkin (KMO) value for validity is 0.955 and 0.930, respectively, indicating that the questionnaire has a good reliability and validity. Finally, the confirmatory factor analysis (CFA) was conducted to determine the validity of educational situation perception as an entire scale. Satisfactory model fits were found with χ^2^/df = 3.423, TuckerLewis index (TLI) = 0.938, comparative fit index (CFI) =0.957, root mean square error of approximation (RMSEA) = 0.078, and standardized root mean residual (SRMR) = 0.046.

#### Teacher support

The scale of Teacher Support was adapted from Lai (2015), which was examined and proved to be valid. The scale contained 7 items, involving affection support (2 items), capacity support (2 items), and behavior support (3 items). A sample item is “My language teacher discussed with us how technological resources or tools could enhance language learning.” Participants rated the degree of conformity with their perceptions of teacher support using a six-point Likert scale, ranging from 1 (strongly disagree) to 6 (strongly agree). The SFLs of the 7 items range from 0.806 to 0.848, the Cronbachαvalue is 0.888, and the KMO value for validity is 0.851, indicating that the scale has a good reliability and validity.

#### Online learning engagement

Schaufeli et al.’s (2002) eight-item Learning Engagement Scale was revised to fit the study context. A sample item is “I was willing to spend time learning English on the network platform.” Participants rated the degree of conformity with their actual learning situation on a 6-point Likert scale ranging from 1 = very inconsistent to 6 = very consistent. Higher scores indicate higher engagement in learning. As the SFLs of the eight items range from 0.807 to 0.868, Cronbach’s α is 0.945, and the KMO value for validity is 0.930, the scale had good reliability.

#### Academic self-efficacy

This study adopted the five-item Academic Self-Efficacy Scale developed by Greene et al. (2004) to measure the degree of confidence that students have in coping with language learning challenges. The wording of items was modified for the current study so that items were anchored to a university context (e.g., “I felt confident that I can learn college English well in technology-based environments.”). Participants rated items on a 6-point Likert scale ranging from 1 (strongly disagree) to 6 (strongly agree). Higher scores indicate higher academic self-efficacy in learning. Greene et al. (2004) reported adequate internal consistency reliability for the scale (α = 0.91), and in the current sample, the internal consistency reliability of this scale was also good, as the SFLs of the 5 items range from 0.853 to 0.936, Cronbach’s α is 0.941, and the KMO value for validity is 0.892.

#### Method of data analysis

The descriptive statistics were conducted by SPSS21.0 to examine all free parameters of the four variables for statistical significance. Besides, in this study, structural equation modeling was used, and a two-stage approach to data analysis was adopted (Anderson and Gerbing, 1988). The first step is to analyze the measurement model, which defines the relationship between the latent structure and the observed measurement factors. The second step is to analyze the structural model, which specifically defines the relationship among latent structures. Amos 21.0 was used to analyze the model, and a variance–covariance matrix as input and maximum likelihood as the method for estimation was adopted. Several fitting indices were used to evaluate the overall model fit.

## Results

### Descriptive statistics

Despite the previous research concerning gender differences was scarce, this study assumed that there may be gender differences in adopting technology to pursue knowledge through network platform. Therefore, independent samples T-test was used to determine whether there were gender differences among the four constructs. The results (see Table 1) indicated that the participants’ gender did not significantly correlate with the four constructs (*p* > 0.05).

**Table 1:** Independent samples *t*-test.

	gender	N	Mean	t	Sig.
ESP	Male	126	4.499	0.218	0.828
	Female	266	4.474		
TS	Male	126	4.522	0.569	0.570
	Female	266	4.459		
OLE	Male	126	4.507	1.527	0.127
	Female	266	4.328		
ASE	Male	126	4.519	0.702	0.483
	Female	266	4.421		

ESP = educational situation perception; TS = teacher support; OLE = online learning engagement; ASE = academic self-efficacy.

Table 2 showed the examining results of the mean, standard deviation, skewness, and kurtosis of all the 34 items. All mean scores were far above the mid-point of 3.5, indicating participants’ positive response to the variables in the questionnaire. The standard deviations ranged from 0.713 to 0.982, which was indicative of a narrow spread of participants’ responses. Skewness and kurtosis indices were within the recommended level of [3] and [10], respectively (Kline, 2005), showing the presence of univariate normality. All the measures had acceptable reliabilities (Cronbach α ranged from 0.888 to 0.955).

**Table 2:** Descriptive statistics of the study constructs.

Constructs	Items	Mean	SD	Cronbach α	Skewness	Kurtosis
ESP	14	4.482	0.857	0.955	−0.551	0.128
TS	7	4.479	0.713	0.888	−0.455	0.088
OLE	8	4.385	0.788	0.945	−0.455	0.049
ASE	5	4.453	0.982	0.941	−0.590	−0.353

Pearson correlation matrices for the relations between variables displayed in Table 3 indicated that there are significant correlations among the study variables. But none of the correlation coefficients exceeded 0.80, excluding the issue of multicolinearity (Tabachnick and Fidell, 2007). Definitely, ESP was significantly and positively correlated to TS (*r* = 0.792, *p* < 0.01), OLE (*r* = 0.719, *p* < 0.01), and ASE (*r* = 0.479, *p* < 0.01). TS was significantly and positively correlated to OLE (*r* = 0.780, *p* < 0.01), and ASE (*r* = 0.540, *p* < 0.01). OLE was significantly and positively correlated with ASE (*r* = 0.591, *p* < 0.01). These results supported the research hypotheses of this study. To further examine the research hypotheses, the following model analyses were conducted to be linked with the above correlations of variables.

**Table 3:** Discriminant validity for the measurement model.

Constructs	ESP	TS	OLE	ASE
ESP	(0.858)			
TS	0.792**	(0.837)		
OLE	0.719**	0.780**	(0.850)	
ASE	0.479**	0.540**	0.591**	(0.901)

*N* = 392. Diagonal in parentheses: square root of average variance extracted from observed variables (items); and off-diagonal: correlations between constructs.

* *p* < 0.01.

### Test of the measurement model

This study used Amos 21.0 with Maximum Likelihood Estimation to analyze the models and estimate parameters, including the procedures of assessing the reliability of items and variables, the convergent and discriminant validity, the path coefficients and the model predicative power. By Hair Jr. et al. (2010), Cronbach’s alpha, composite reliability (CR) and average variance extracted (AVE) were considered as the main criteria for examining reliability and convergent validity. “Convergent validity, which examines whether individual indicators are indeed measuring the constructs they are purported to measure, was assessed using standardized indicator factor loadings, and they should be significant and exceed 0.7, and average variance extracted (AVE) by each construct should exceed the variance due to measurement error for that construct (i.e., AVE should exceed 0.50)” (Teo and van Schaik, 2012, p. 182). The results of the data analysis in this study indicated that the SFL of all items of the constructs exceeded the minimum value of 0.70, and the AVE values ranged from 0.701 to 0.811, far higher than the threshold value of 0.50. Hence, this measurement model in this study established the convergent validity of all the measurement items. Table 3 indicated that the square root of AVE (shown in parentheses along the diagonal) of each construct was higher (0.837 to 0.901) than corresponding correlation values for that variable in all cases, thereby assuring discriminant validity.

In addition, the model fit was tested by the normed X^2^ statistics (X^2^/df), the root mean square error of approximation (RMSEA), Standardized Root Mean Residual (SRMR), the comparative fit index (CFI), and the incremental index (TLI). According to the results of data analysis, there was adequate model fit for the measurement model, X^2^/df = 2.665, TLI = 0.953, CFI = 0.967, RMSEA = 0.071, and SRMR = 0.047, indicating that the items were reliable indicators of the hypothesized constructs, thus allowing tests of the structural relationships in the various models to proceed (Teo and van Schaik, 2012).

### Test of the structural model

Following the recommendations by Hu and Bentler (1999), the model fit was tested by using several goodness-off it indexes, including the ratio of the chi-square to its degrees of freedom (X^2^/df), RMSEA, SRMR, CFI, and TLI. By Hair Jr. et al. (2010), values of X^2^/df (<3), CFI (>0.90), TLI (>0.90), RMSEA (<0.08), and SRMR (<0.08) are reflective of a good fit.

**Figure 1:**
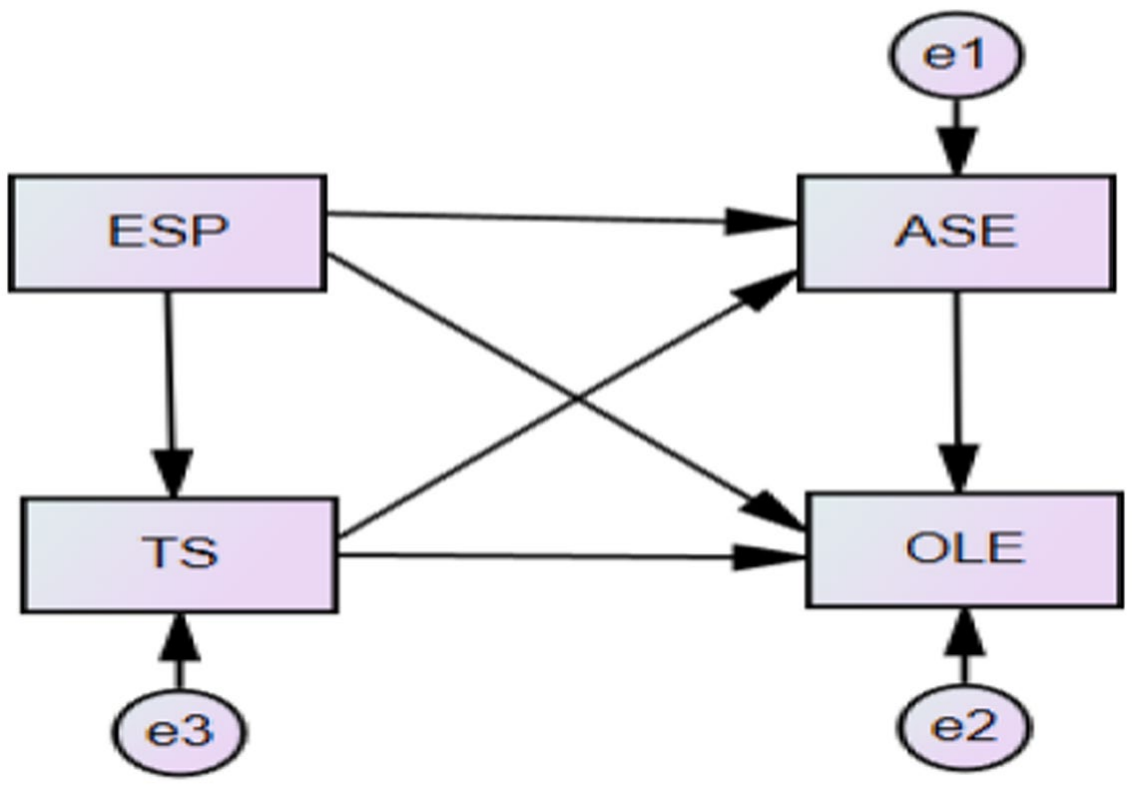
The hypothesized research model.

**Figure 2:**
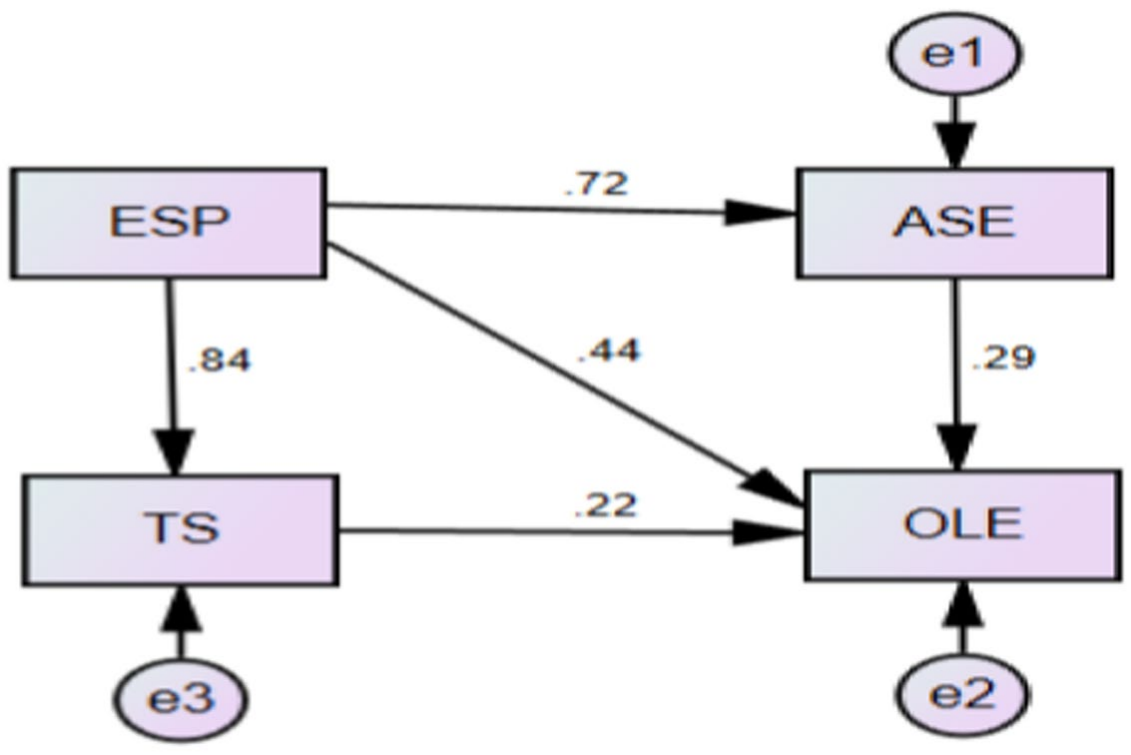
The final test model.

The hypothesized research model (Figure 1) was then tested, and was found that the saturated model was not fitted to the data of at least one group. For this reason, only the ‘function of log likelihood’, AIC and BCC are reported. The likelihood ratio chi square statistic and other fit measures are not reported. After deleting the path (TS → ASE with a path coefficient of 0.17, *p* < 0.05, showing a significant effect of TS on ASE, which is consistent with the previous study of Pan and Chen (2021) concerning this perspective), the final model (Figure 2) was constructed and was found to have good mode fit indices with X^2^/df = 2.936, CFI = 0.995, TLI = 0.971, SRMR = 0.023, and RMSEA = 0.076.

The association between ESP and TS was vitally significant [β = 0.84, *p* < 0.001, and 95%CI (0.77, 0.90)]. Similarly, ESP was positively predictive of ASE [β = 0.72, *p* < 0.001, and 95%CI (0.63, 0.80)]. Also, ESP was significantly associated with OLE [β = 0.44, *p* < 0.001, and 95%CI (0.29, 0.59)]. ASE was significantly associated with OLE [β = 0.29, *p* < 0.001, and 95%CI (0.21, 0.39)]. The association of TS with OLE was significant [β = 0.22, *p* < 0.001, and 95%CI (0.08, 0.37)], and meanwhile TS mediated the relationship between ESP and OLE with a significant effect [β = 0.23, *p* < 0.001, and 95%CI (0.11, 0.36)], which indicated that TS not only directly correlated with OLE but also played a vital mediational role. Besides, the relationship between ESP and OLE, mediated by ASE, showed a significant effect [β = 0.39, *p* < 0.001, and 95%CI (0.27, 0.52)], which indicated the vitally important role that ASE played when ESP exerted effects on OLE. Taken together, the results demonstrated that TS and ASE played significant mediational roles in the multivariate relationships, which corroborated the previous research on the significance of teacher support and the effect of psychological needs.

### Mediation analysis

The mediation effect was detected using bootstrapping test with structural equation model (Cheung and Lau, 2008). From Figure 2, ASE mediated the relationship between ESP and OLE, and TS mediated the relationship between ESP and OLE. The summary of the mediation analysis shown in Table 4 indicated statistically significance and accorded with the guidelines by Cohen (1988) with medium (0.1–0.5) indirect effect values.

**Table 4:** Summary of mediation analysis.

From	β	Mediator	β	To	Indirect effect	95% confidence interval
ESP	0.72	ASE	0.29	OLE	0.39***	[0.27–0.52]
ESP	0.84	TS	0.22	OLE	0.23***	[0.11–0.36]

*N* = 392. (Bootstrapping test). Number of Bootstrap samples = 5000.

*** *p* < 0.001.

## Discussion

The present study addressed the roles of educational situation perception, teacher support and academic self-efficacy in Chinese emerging adults’ online learning engagement in university studies. The research findings contribute to better understanding of young Chinese university students’ online learning engagement in technology-based language learning in several ways. First, educational situation perception and teacher support are related to and facilitate students’ online learning engagement. Second, academic self-efficacy was significantly associated with the increase in online learning engagement. Third, academic self-efficacy and teacher support serve as mediating variables between educational situation perception and online learning engagement.

### Universal influences of educational situation perception

Consistent with previous studies, the research results confirmed the adaptive benefits of educational situation perception on online learning engagement, the influence on academic self-efficacy, and the association with teacher support in technology-based language learning (Ryan and Deci, 2000; Shadiev et al., 2020). This study found that educational situation perception significantly correlated with students’ online learning engagement, thus verifying the previous research which highlighted the significance of educational situation perception in promoting students’ perception of online learning environments, sense of participation, learning satisfaction, and positive outcomes (Saini and Goel, 2019; Zhao et al., 2014). The findings also confirmed previous researchers’ assertion that educational situation is an accurate representation of the existing state, evolution mechanism and interaction relationship of the entity factors such as human, machine, and environment under the real learning space–time condition, the function of which is to trigger the different dimensions of learning engagement (Lu et al., 2022) and support the learning process (Xie et al., 2019). As such, educational situation perception can help enhance students’ technology-enhanced learning experience and promote trust among group members, an important precondition for computer-supported collaborative learning (Gerdes, 2010). This study highlighted the direct association of educational situation perception with online learning engagement in technology-enhanced language learning, and meanwhile examined the mediational effect of teacher instructional behaviors (e.g., teacher supports) that may accelerate students’ language learning with technology (Reinders, 2010). The results of this study are a useful supplement and expansion of previous studies, as some scholars have put forward an educational situation perception model (Cukurova et al., 2019; Gu et al., 2019) from the collaborative perspectives of “human, machine, object and environment,” aiming at the practical needs of the classroom teaching reform enabled by intelligent technology, the purpose of which is to quantitatively analyze the various components of the intelligent learning space, and to provide empirical support for constructing language learning in a technological environment. Despite that this study examined the influence of educational situation perception on teacher support, future studies may conduct more in-depth exploration on how the perception of educational situation could be formed through different types of cognitive and metacognitive support that teachers provide.

These findings echoed the critical role of computer-supported collaborative learning environment in affecting learning behavior engagement (Hernandez-Selles et al., 2019), as the authentic educational situations perceived by the online learners assist constituting “a sense of ‘realness’, a quality of not being fake or contrived” (Rambe and Mkono, 2018, p. 704). Effective network environment and supportive network systems help to improve the level of behavior engagement of language learners, which is consistent with the existing research conclusion that “social presence was found to evolve from interaction, and an optimal level of social presence encouraged participation and positively shaped the dynamics of interaction, and thereby promoted collaboration” (Zhao et al., 2014, p. 817). The significant function of educational situation perception found in this study corroborated the empirical research from Redmond and Lock (2006) which identified that real context and harmonious e-learning atmosphere help to establish appropriate teaching situations and improve students’ learning participation. The research of Koranteng et al. (2018) demonstrated that the supportive network system environment had a positive impact on learners’ continuous behavioral engagement and the achievement of synergistic interaction led to deeper learning.

This finding also suggested the mediational function of active teacher support to bridge collaborative learning environment and learners’ actual expectations. Thereby, this study highlighted the following three aspects. The first is to strengthen the consideration of the key elements of the educational situation. With the gradual advancement of the scientific research on learning, an increasing number of scholars have paid attention to the research on the mechanism of the influence of the presentation of teaching resources, the organizational mode of teaching activities, the teaching behaviors, and teaching styles of teachers on the learners’ learning process and outcomes. Meanwhile, much more attention was paid to the influence of the interaction mechanism between learners and teachers, teaching content and teaching resources on technology-based self-directed learning. Therefore, the development of the research on educational situation perception needs a comprehensive consideration of the entity elements that constitute the complete educational context from the level of data perception, and the use of intelligent perception technology, to realize the comprehensive evaluation of students, teachers, teaching resources, teaching media, teaching environment and other elements of the state of existence and evolution model, and to achieve the accurate representation of the complete education situation. The second is to clarify the key position of intelligent technology in educational situations. With the deepening of the integration of intelligent technology and teaching, the future learning space will present the developmental trend of ubiquitous intelligence, virtual-real integration and man–machine cooperation (Gu et al., 2019), and the entity of intelligent education will occupy a more and more important key position in the future learning space through the creation of intelligent learning environment, the reconstruction of intelligent analysis method and the overall optimization of educational situations. And the third is to emphasize the reconstruction of intelligence teaching ecology driven by learning activities. According to the activity theory, the achievement of educational goal is the result of individual and group synergy (Engeström, 2001), and the interaction between elements under the network technology environment can be effectively implemented through the development of learning activities. Thus, it is necessary to emphasize the key role of intelligent learning activities in the construction of the whole educational situation, and to strengthen the deconstruction of activity-based teaching process, so as to realize the multidimensional integration of the whole educational context.

### Teacher support in online learning engagement

From the results, teacher support directly influenced online learning engagement. This is consistent with the previous research which have reported that the guidance and support from teachers drove students’ engagement in technology-based self-directed language learning (Ertmer, 2005), helped students incorporate learning resources/activities into their learning ecology (Lai et al., 2016), and facilitated students to utilize technology as learning tools (McLoughlin and Lee, 2010). Similarly, Skinner and Pitzer’s (2012) research concerning the multilevel aspects on online learning engagement demonstrated the need for a theoretically driven, psychometrically sound scale to measure learner engagement in technology-enhanced learning environments from the perspective of teacher support (Deng et al., 2020). Practically, students’ online learning engagement in college English course in Chinese universities, to a great extent, builds on their self-directed learning behaviors beyond the classroom. Therefore, this study results suggested that teachers provide support for students’ self-directed learning behaviors in technology-based language learning environments. Autonomy support is an important concept in self-determination theory and a new perspective in current positive psychology research. From the teacher’s point of view, it is also understood as a kind of motivation tendency or style, which is opposite to the “controlling” style or tendency, to promote students’ learning and development in a way that supports students’ self-directed learning motivation. Thus, self-supporting teaching model has become a direction of teaching model transformation, as teachers’ conducting self-supporting teaching can significantly promote the play of classroom functions and enhance the effectiveness of technology-based online teaching and learning.

In addition, this study revealed the pathway that educational situation perception influenced online learning engagement indirectly through teacher support, which extends our understanding of students’ growth and development through teacher support on the journey of technology-enhanced learning experience. Meanwhile, this finding corroborates the previous research that teachers significantly shape the quality of students’ learning experiences by affecting students’ cognitive, affective and social learning behaviors (Farmer et al., 2011) and, as a significant social agent, teachers play a critical role in helping students develop autonomy of technology-based language learning beyond class (Reinders and Darasawang, 2012). In combination with the above research conclusions, this study holds that the Education Administrative Department should coordinate with the school to promote the construction of the mechanism of teacher professional development, enhance the support willingness and capacity of the teachers, and provide the policy guarantee and the intelligence support for the promotion of the teacher support level to the student. On the other hand, this study puts forward the following suggestions for the future development of online teaching: (1) to optimize online teaching design and pay attention to the challenge, authenticity and interest of teaching tasks, as the design of online learning activities is a key factor to ensure the effectiveness of online learning; (2) to promote the feedback literacy of teachers and students, as the multidimensional interactive feedback between teachers, students, and technological environments is helpful to construct effective teaching dialog and promote students’ in-depth learning; and (3) to carry out innovative teaching practice of technical empowerment and promote the effective integration of online and offline teaching. In the post-epidemic period, teachers should make full use of the unique advantages of online and offline teaching and actively develop blended teaching design. Online learning endows learners with more autonomy, and teachers can encourage learners to make full use of online learning resources to develop interest-driven knowledge constructive learning. Immersive virtual reality environment can provide real-time interactive feedback for learners, and help to increase the degree of learning engagement of learners.

### Direct and mediating effects of academic self-efficacy

This study found that academic self-efficacy directly influenced online learning engagement. This result is consistent with that of most literature studies. This study indicated that academic self-efficacy has a greater impact on students’ online learning involvement than teacher’s support, which indicates that academic self-efficacy is one of the social psychological constructs highly related to online learning involvement. Students’ efficacy beliefs are the internal factors that motivate students’ positive behaviors, so they have a more direct and strong influence on online learning investment. One explanation for this is that when students are more confident, they tend to show greater self-control, and when faced with failure, they work harder and achieve better grades. Besides, the mediating role of academic self-efficacy in the association between educational situation perception and online learning engagement in technology-based environments has been widely researched (Ratelle et al., 2005; Joussemet et al., 2008; Zuffianò et al., 2013). Consistent with previous research, this study found that academic self-efficacy plays a significant mediating role between educational context perception and online learning engagement. The mediating effect of academic self-efficacy indicates that educational situation perception can enhance students’ academic self-efficacy and promote online learning engagement. According to self-determination theory, the perception of educational context can satisfy three basic needs of students, especially the need for relationship, thus helping students to develop and enhance their academic self-efficacy. According to Yesilyurt et al. (2016), academic self-efficacy is an intermediate variable between perceived interpersonal environment and active learning engagement. Therefore, this study held that it is necessary to strengthen students’ psychological and behavioral training to enhance students’ academic self-efficacy. In particular, teachers need to strengthen the guidance to facilitate students to actively cultivate the sense of academic self-efficacy. Specifically, teachers should help students to establish clear and specific learning goals, so that students have more successful online learning experience. Teachers should provide reasonable role models for students to enhance effective alternative experience and strengthen the attribution guidance, so that students learn to achieve active self-attribution.

### Limitations and future directions

This study was not without limitations, which could set avenues for future research. Firstly, the simplex cross-sectional design being applied in this study may result in a common method bias (Teo and Noyes, 2014). For instance, although previous research demonstrated that educational situation perception contributed to various positive achievement-relevant outcomes for students, such as higher academic performance, sense of learning achievement, stronger intrinsic motivation, and self-efficacy (Jelfs and Whitelock, 2000; Kahrimanis et al., 2011; Blau et al., 2019), suggesting a pathway from educational situation perception to students’ online learning engagement; it is still possible that students’ demonstration of online learning engagement might be susceptible to multiple variables (e.g., individual attitude and motivation). Hence, it is suggested that future study adopt multilayered, multidimensional methods (e.g., the combination of cross-sectional design with longitudinal research) to enhance our understanding of the causality as far as possible.

Second, this study focused on the association of educational situation perception with online learning engagement. Although previous studies demonstrated that educational situation perception can be predictive of students’ learning endeavor behaviors (de Barba et al., 2016), the different teaching leadership of educators, the diversity of students’ learning styles and the limitations of network hardware environment may also lead to some differences in the research results. Future research could benefit from investigating how definite environmental elements could influence students’ achievement behaviors in language learning and its potential mechanism.

Third, this study did not form an effective measurement of interactive mechanisms of online learning engagement. With the development of media-based English learning, other potential factors, such as new media literacy, teacher or peer feedback, can be incorporated into the follow-up study to construct a more comprehensive influence mechanism model of online learning engagement. In the future, multimodal data supported by brain science and artificial intelligence can be used to accurately measure and synthetically analyze the online learning engagement, and strengthen the real-time tracking and monitoring of the learners’ online learning behaviors.

## Conclusion

Overall, the results of the current research provided evidence of the association between educational situation perception, teacher support, online learning engagement, and academic self-efficacy. The data supported the major hypotheses of educational situation perception’s influence on online learning engagement, and this effect was found to be mediated by academic self-efficacy and teacher support. This research extended our understanding of the consequences of educational situation perception by investigating its influences on students’ experience of technology-based language learning. The results of such study would inform teacher educators and network administrators for curriculum and technological development purposes. Finally, in consideration of globally pervasive technology use in educational landscapes and the complexity of online learning, cross-cultural comparative studies could be conducted to identify the culture-invariant variables that influence students’ educational situation perception and online learning engagement.

## Data availability statement

The original contributions presented in the study are included in the article/supplementary material, further inquiries can be directed to the corresponding author.

## Ethics statement

Ethical review and approval was not required for the study on human participants in accordance with the local legislation and institutional requirements. The patients/participants provided their written informed consent to participate in this study.

## Author contributions

The author confirms being the sole contributor of this work and has approved it for publication.

## Funding

This work was supported by the Research Project of the 2021 Teaching Reform of Xingzhi College, Zhejiang Normal University (Grant no. ZC303921073).

## Conflict of interest

The author declares that the research was conducted in the absence of any commercial or financial relationships that could be construed as a potential conflict of interest.

## Publisher’s note

All claims expressed in this article are solely those of the authors and do not necessarily represent those of their affiliated organizations, or those of the publisher, the editors and the reviewers. Any product that may be evaluated in this article, or claim that may be made by its manufacturer, is not guaranteed or endorsed by the publisher.

## References

[ref1] Affuso G. Bacchini D. Miranda M. C. (2017). The contribution of school-related parental monitoring, self-determination, and self-efficacy to academic achievement. J. Educ. Res. 110, 565–574. doi: 10.1080/00220671.2016.1149795

[ref2] Aleven V. McLaughlin E. A. Glenn R. A. Koedinger K. R. (2017). “Instruction based on adaptive learning technologies,” in Handbook of research on learning and instruction. eds. Mayer R. E. Alexander P. A. (New York, NY: Routledge), 522–560.

[ref3] Alivernini F. Lusidi F. (2011). Relationship between social context, self-efficacy, motivation, academic achievement, and intention to drop out of high school: a longitudinal study. J. Educ. Res. 104, 241–252. doi: 10.1080/00220671003728062

[ref4] Anderson J. C. Gerbing D. W. (1988). Structural equation modeling in practice: a review and recommended two-step approach. Psychol. Bull. 103, 411–423. doi: 10.1037/0033-2909.103.3.411

[ref5] Bandura A. (1997). Self-efficacy: The exercise of control. New York: Freeman.

[ref6] Bandura A. (2001). Social foundations of thought and action: A social cognitive theory. Upper Saddle River: Prentice Hall.

[ref7] Barron B. (2004). Learning ecologies for technological fluency in a technology-rich community. J. Educ. Comput. Res. 31, 1–36. doi: 10.2190/1N20-VV12-4RB5-33VA

[ref8] Bassi M. Steca P. Delle Fave A. Caprara G. V. (2007). Academic self-efficacy beliefs and quality of experience in learning. J. Youth Adol. 36, 301–312. doi: 10.1007/s10964-006-9069-y, PMID: 27519029

[ref9] Benson P. Reinders H. (2011). Beyond the language classroom. New York, NY: Palgrave Macmillan.

[ref10] Blau I. Shamir-Inbal T. Avdiel O. (2019). How does the pedagogical design of a technology-enhanced collaborative academic course promote digital literacies, self-regulation, and perceived learning of students? Internet High. Educ. 45:100722. doi: 10.1016/j.iheduc.2019.100722

[ref11] Bligh B. Crook C. (2017). “Learning spaces,” in Technology enhanced learning: Research themes. eds. Duval E. Sharples M. Sutherland R. (Cham, Switzerland: Springer), 69–88.

[ref12] Bower M. Vlachopoulos P. (2018). A critical analysis of technology-enhanced learning design frameworks. Br. J. Educ. Technol. 49, 981–997. doi: 10.1111/bjet.12668

[ref13] Carson L. Mynard J. (2012). “Introduction” in Advising in language learning: Dialogue, tools and context. eds. Mynard J. Carson L. (Harlow: Pearson Education Limited), 3–25.

[ref14] Castañeda L. Selwyn N. (2018). More than tools? Making sense of the ongoing digitizations of higher education. Int. J. Educ. Technol. High. Educ. 15, 1–10. doi: 10.1186/s41239-018-0109-y

[ref15] Chau K. Y. Law K. M. Y. Tang Y. M. (2021). Impact of self-directed learning and educational technology readiness on synchronous E-learning. J. Organ. End User Comp. 33, 1–20. doi: 10.4018/JOEUC.20211101.oa26

[ref16] Cheng X. Li Y. Sun J. Huang J. (2016). Application of a novel collaboration engineering method for learning design: a case study. Br. J. Educ. Technol. 47, 803–818. doi: 10.1111/bjet.12382

[ref04] Cheung G. W. Lau R. S. (2008). Testing mediation and suppression effects of latent variables: Bootstrapping with structural equation models. Organ. Res. Methods 11, 296–325. doi: 10.1177/1094428107300343

[ref17] Chiu T. K. (2021). Digital support for student engagement in blended learning based on self-determination theory. Comput. Hum. Behav. 124:106909. doi: 10.1016/j.chb.2021.106909

[ref18] Chu H. C. Hwang G. J. Tsai C. C. (2010). A knowledge engineering approach to developing mindtools for context-aw ubiquitous learning. Comput. Educ. 54, 289–297. doi: 10.1016/j.compedu.2009.08.023

[ref05] Cohen J. (1988). Statistical power analysis for the behavioral sciences, 2nd Edn. Hillsdale: Erlbaum.

[ref19] Coll C. Rochera M. J. de Gispert I. (2014). Supporting online collaborative learning in small groups: teacher feedback on learning content, academic task and social participation. Comput. Educ. 75, 53–64. doi: 10.1016/j.compedu.2014.01.015

[ref20] Cui Y. Zhao G. Zhang D. (2022). Improving students’ inquiry learning in web-based environments by providing structure: does the teacher matter or platform matter? Br. J. Educ. Technol. 53, 1049–1068. doi: 10.1111/bjet.13184

[ref21] Cukurova M. Kent C. Luckin R. (2019). Artificial intelligence and multimodal data in the service of human decision-making: a case study in debate tutoring. Br. J. Educ. Technol. 50, 3032–3046. doi: 10.1111/bjet.12829

[ref014] de Barba P. G. Kennedy G. E. Ainley M. D. (2016). The role of students’ motivation and participation in predicting performance in a MOOC. J. Comput. Assist. Learn. 32, 218–231. doi: 10.1111/jcal.12130

[ref22] De Corte E. (2012). Constructive, self-regulated, situated, and collaborative learning: an approach for the acquisition of adaptive competence. J. Educ. 192, 33–47. doi: 10.1177/0022057412192002-307

[ref23] Deci E. L. Ryan R. M. (2000). The “what” and “why” of goal pursuits: human needs and the self-determination of behavior. Psychol. Inq. 11, 227–268. doi: 10.1207/S15327965PLI1104_01

[ref24] Deng R. Benckendorff P. Gannaway D. (2020). Learner engagement in MOOCs: scale development and validation. Br. J. Educ. Technol. 51, 245–262. doi: 10.1111/bjet.12810

[ref25] Dessi D. Fenu G. Marras M. Recupero D. R. (2019). Bridging learning analytics and cognitive computing for big data classification in micro-learning video collections. Comput. Hum. Behav. 92, 468–477. doi: 10.1016/j.chb.2018.03.004

[ref01] Dey A. (2001). Understanding and Using Context. Pers. Ubiquitous Comput. 5, 4–7. doi: 10.1007/s007790170019

[ref26] Engeström Y. (2001). Expansive learning at work: toward an activity theoretical reconceptualization. J. Educ. Work. 14, 133–156. doi: 10.1080/13639080020028747

[ref27] Ertmer P. A. (2005). Teacher pedagogical beliefs: the final frontier in our quest for technology integration? Educ. Technol. Res. Dev. 53, 25–39. doi: 10.1007/bf02504683

[ref28] Fang F. M. Zhang L. (2012). Teachers’ roles in promoting students’ learner autonomy in China. Engl. Lang. Teach. 5, 51–56. doi: 10.5539/elt.v5n4p51

[ref29] Farmer T. W. Lines M. M. Hamm J. V. (2011). Revealing the invisible hand: the role of teachers in children's peer experiences. J. Appl. Dev. Psychol. 32, 247–256. doi: 10.1016/j.appdev.2011.04.006

[ref30] Gallivan M. J. Spitler V. K. Koufaris M. (2005). Does information technology training really matter? A social information processing analysis of coworkers’ influence on IT usage in the workplace. J. Manag. Inf. Syst. 22, 153–192. doi: 10.1080/07421222.2003.11045830

[ref31] García Botero G. Questier F. Zhu C. (2019). Self-directed language learning in a mobile-assisted, out-of-class context: do students walk the talk? Comput. Assist. Lang. Learn. 32, 71–97. doi: 10.1080/09588221.2018.1485707

[ref32] Garrison D. R. Akyol Z. (2009). Role of instructional technology in the transformation of higher education. J. Comput. High. Educ. 21, 19–30. doi: 10.1007/s12528-009-9014-7

[ref33] Gawande V. Al-Senaidi S. (2015). Situated learning: learning in a contextual environment. Int. J. Comp. Acad. Res. 4, 207–213.

[ref34] Gerdes A. (2010). Revealing preconditions for trustful collaboration in CSCL. Int. J. Comput.-Support. Collab. Learn. 5, 345–353. doi: 10.1007/s11412-010-9090-8

[ref35] Glover I. Hepplestone S. Parkin H. J. Rodger H. Irwin B. (2016). Pedagogy first: Realising technology enhanced learning by focusing on teaching practice. Br. J. Educ. Technol. 47, 993–1002. doi: 10.1111/bjet.12425

[ref36] Goodyear P. Jones C. Thompson K. (2014). “Computer-supported collaborative learning: instructional approaches, group processes and educational designs,” in Handbook of research on educational communications and technology. eds. Spector J. M. Merrill M. D. Elen J. Bishop M. J. (New York: Springer), 439–451.

[ref37] Greene B. A. Miller R. B. Crowson H. M. Duke B. L. Akey K. L. (2004). Predicting high school students’ cognitive engagement and achievement: contributions of classroom perceptions and motivation. Contemp. Educ. Psychol. 29, 462–482. doi: 10.1016/j.cedpsych.2004.01.006

[ref38] Gu X. Crook C. Spector M. (2019). Facilitating innovation with technology: key actors in educational ecosystems. Br. J. Educ. Technol. 50, 1118–1124. doi: 10.1111/bjet.12786

[ref39] Hair J. F. Jr. Black W. C. Babin B. J. Anderson R. E. (2010). Multivariate data analysis: A global perspective, 7th Edn. London: Pearson

[ref40] He T. Li S. (2019). A comparative study of digital informal learning: the effects of digital competence and technology expectancy. Br. J. Educ. Technol. 50, 1744–1758. doi: 10.1111/bjet.12778

[ref41] Healey M. Jordan F. Pell B. Short C. (2010). The research-teaching nexus: a case study of students’ awareness, experiences and perceptions of research. Innov. Educ. Teach. Int. 47, 235–246. doi: 10.1080/14703291003718968

[ref09] Hernandez-Selles N. Munoz-Carril P.-C. Gonzalez-Sanmamed M. (2019). Computer-supported collaborative learning: An analysis of the relationship between interaction, emotional support and online collaborative tools. Comput. Educ. 138, 1–12. doi: 10.1016/j.compedu.2019.04.012

[ref42] Hew K. F. (2016). Promoting engagement in online courses: what strategies can we learn from three highly rated MOOCS. Br. J. Educ. Technol. 47, 320–341. doi: 10.1111/bjet.12235

[ref43] Hu L. T. Bentler P. M. (1999). Cutoff criteria for fit indexes in covariance structure analysis: conventional criteria versus new alternatives. Struct. Equ. Model. 6, 1–55. doi: 10.1080/10705519909540118

[ref44] Hughes J. N. Chen Q. (2011). Reciprocal effects of student-teacher and student-peer relatedness: effects on academic self-efficacy. J. Appl. Dev. Psychol. 32, 278–287. doi: 10.1016/j.appdev.2010.03.005, PMID: 21927528 PMC3173774

[ref45] Hughes J. N. Luo W. Kwok O. M. Loyd L. K. (2008). Teacher-student support, effortful engagement, and achievement: a 3-year longitudinal study. J. Educ. Psychol. 100, 1–14. doi: 10.1037/0022-0663.100.1.1, PMID: 19578558 PMC2705122

[ref46] Hung M. L. Chou C. Chen C. H. Own Z. Y. (2010). Learner readiness for online learning: scale development and student perceptions. Comp. Educ. 55, 1080–1090. doi: 10.1016/j.compedu.2010.05.004

[ref47] Hung P.-H. Hwang G.-J. Lin Y.-F. Wu T.-H. Su I. H. (2013). Seamless connection between learning and assessment: applying progressive learning tasks in Mobile ecology inquiry. Educ. Technol. Soc. 16, 194–205.

[ref48] Hwang G. J. Chang H. F. (2011). A formative assessment-based mobi1e learning approach to improving learning attitudes and achievements of students. Comput. Educ. 56, 1023–1031. doi: 10.1016/j.compedu.2010.12.002

[ref49] Inozu J. Sahinkarakas S. Yumru H. (2010). The nature of language learning experiences beyond the classroom and its learning outcomes. US-China Foreign Language 8, 14–21.

[ref50] Jelfs A. Whitelock D. (2000). The notion of presence in virtual learning environments: what makes the environment “real”. Br. J. Educ. Technol. 31, 145–152. doi: 10.1111/1467-8535.00145

[ref51] Jeno L. M. Adachi P. J. C. Grytnes J. Vandvik V. Deci E. L. (2019). The effects of m-learning on motivation, achievement and well-being: a self-determination theory approach. Br. J. Educ. Technol. 50, 669–683. doi: 10.1111/bjet.12657

[ref013] Joussemet M. Landry R. Koestner R. (2008). A self-determination theory perspective on parenting. Can. Psychol. 49, 194–200. doi: 10.1037/a0012754

[ref52] Jung Y. Lee J. (2018). Learning engagement and persistence in massive open online courses (MOOCS). Comput. Educ. 122, 9–22. doi: 10.1016/j.compedu.2018.02.013

[ref53] Kahrimanis G. Avouris N. Komis V. (2011). “Interaction analysis as a tool for supporting collaboration: an overview,” in Technology-enhanced systems and tools for collaborative learning scaffolding. eds. Daradoumis T. Caballé S. Juan A. Xhafa F. (Berlin Heidelberg: Springer), 93–114.

[ref54] Kearsley G. Shneiderman B. (1998). Engagement theory: a framework for technology-based teaching and learning. Educ. Technol. 38, 20–23.

[ref55] Kennedy C. Miceli T. (2010). Corpus-assisted creative writing: introducing intermediate Italian learners to a corpus as a reference resource. Lang. Learn. Technol. 14, 28–44.

[ref56] Klem A. Connell J. (2004). Relationships matter: linking teacher support to student engagement and achievement. J. Sch. Health 74, 262–273. doi: 10.1111/j.1746-1561.2004.tb08283.x, PMID: 15493703

[ref03] Kline R. B. (2005). Principles and practice of structural equation modeling (2nd Edn). New York: Guilford.

[ref57] Koranteng F. N. Wiafe I. Kuada E. (2018). An empirical study of the relationship between social networking sites and students’ engagement in higher education. J. Educ. Comput. Res. 57, 1131–1159. doi: 10.1177/0735633118787528

[ref58] Kulik J. A. Fletcher J. D. (2016). Effectiveness of intelligent tutoring systems: a meta-analytic review. Rev. Educ. Res. 86, 42–78. doi: 10.3102/0034654315581420

[ref59] Lai C. (2015). Modeling teachers’ influence on learners’ self-directed use of technology for language learning outside the classroom. Comput. Educ. 82, 74–83. doi: 10.1016/j.compedu.2014.11.005

[ref60] Lai C. Gu M. Y. (2011). Self-regulated out-of-class language learning with technology. Comput. Assist. Lang. Learn. 24, 317–335. doi: 10.1080/09588221.2011.568417

[ref61] Lai C. Hu X. Lyu B. (2018). Understanding the nature of learners’ out-of-class language learning experience with technology. Comput. Assist. Lang. Learn. 31, 114–143. doi: 10.1080/09588221.2017.1391293

[ref62] Lai C. Shum M. Tian Y. (2016). Enhancing learners’ self-directed use of technology for language learning: the effectiveness of an online training platform. Comput. Assist. Lang. Learn. 29, 40–60. doi: 10.1080/09588221.2014.889714

[ref63] Lai C. Zheng D. (2018). Self-directed use of mobile devices for language learning beyond the classroom. ReCALL 30, 299–318. doi: 10.1017/S0958344017000258

[ref64] Lam S.-F. Jimerson S. Kikas E. Cefai C. Veiga F. H. Nelson B.. (2012). Do girls and boys perceive themselves as equally engaged in school? The results of an international study from 12 countries. J. Sch. Psychol. 50, 77–94. doi: 10.1016/j.jsp.2011.07.004, PMID: 22386079

[ref65] Law D. C. S. Meyer J. H. F. (2011). Relationships between Hong Kong students’ perceptions of the learning environment and their learning patterns in post-secondary education. High. Educ. 62, 27–47. doi: 10.1007/s10734-010-9363-1

[ref66] Lawless K. A. Pellegrino J. W. (2007). Professional development in integrating technology into teaching and learning: knowns, unknowns, and ways to pursue better questions and answers. Rev. Educ. Res. 77, 575–614. doi: 10.3102/0034654307309921

[ref67] Levy M. (2009). Technologies in use for second language learning. Mod. Lang. J. 93, 769–782. doi: 10.1111/j.1540-4781.2009.00972.x

[ref68] Liyanawatta M. Yang S.-H. Liu Y.-T. Zhuang Y. Chen G. (2021). Audience participation digital drama-based learning activities for situational learning in the classroom. Br. J. Educ. Technol. 53, 189–206. doi: 10.1111/bjet.13160

[ref69] Lu G. Xie K. Liu Q. (2022). What influences student situational engagement in smart classrooms: perception of the learning environment and students’ motivation. Br. J. Educ. Technol. 53, 1665–1687. doi: 10.1111/bjet.13204

[ref70] Malmberg J. Järvelä S. Holappa J. Haataja E. Huang X. Siipo A. (2019). Going beyond what is visible: what multichannel data can reveal about interaction in the context of collaborative learning? Comput. Hum. Behav. 96, 235–245. doi: 10.1016/j.chb.2018.06.030

[ref71] Martin F. Ertzberger J. (2013). Here and now mobile learning: an experimental study on the use of mobile technology. Comput. Educ. 68, 76–85. doi: 10.1016/j.compedu.2013.04.021

[ref72] Mavrikis M. (2010). Modelling student interactions in intelligent learning environments: constructing Bayesian networks from data. Int. J. Artif. Intel. Tools 19, 733–753. doi: 10.1142/S0218213010000406

[ref73] McKenney S. Reeves T. C. (2018). Conducting educational design research. New York, NY: Routledge. 10.1111/medu.14280 PMC7754301 32564385

[ref74] McLoughlin C. Lee M. L. (2010). Personalized and self-regulated learning in the web 2.0 era: international exemplars of innovative pedagogy using social software. Aust. J. Educ. Technol. 26, 28–43. doi: 10.14742/ajet.1100

[ref75] Miao Y. Pinkwart N. Hoppe U. (2006). “Conducting situated learning in a collaborative virtual environment”. in *Proceedings of the Fifth IASTED International Conference on Web-based Education*. ed. Uskov V. (Anaheim, CA: ACTA Press), 7–12.

[ref76] Nevgi A. Virtanen P. Niemi H. (2006). Supporting students to develop collaborative learning skills in technology-based environments. Br. J. Educ. Technol. 37, 937–947. doi: 10.1111/j.1467-8535.2006.00671.x

[ref77] O’Brien H. L. Toms E. G. (2008). What is user engagement? A conceptual framework for defining user engagement with technology. J. Am. Soc. Inf. Sci. Technol. 59, 938–955. doi: 10.1002/asi.20801

[ref78] Olsen J. K. Sharma K. Rummel N. Aleven V. (2020). Temporal analysis of multimodal data to predict collaborative learning outcomes. Br. J. Educ. Technol. 51, 1527–1547. doi: 10.1111/bjet.12982

[ref79] Pan X. Chen W. (2021). Modeling teacher supports toward self-directed language learning beyond the classroom: technology acceptance and technological self-efficacy as mediators. Front. Psychol. 11:564294. doi: 10.3389/fpsyg.2020.564294, PMID: 34975643 PMC8716430

[ref80] Pan X. Shao H. (2020). Teacher online feedback and learning motivation: learning engagement as a mediator. Soc. Behav. Personal. Int. J. 48:e9118. doi: 10.2224/sbp.9118

[ref81] Papachristos N. M. Vrellis I. Natsis A. Mikropoulos T. A. (2013). The role of environment design in an educational multi-user virtual environment. Br. J. Educ. Technol. 45, 636–646. doi: 10.1111/bjet.12056

[ref82] Plass J. Homer B. Kinzer C. (2015). Foundations of game-based learning. Educ. Psychol. 50, 258–283. doi: 10.1080/00461520.2015.1122533

[ref83] Rambe P. Mkono M. (2018). Appropriating WhatsApp-mediated postgraduate supervision to negotiate “relational authenticity” in resource-constrained environments. Br. J. Educ. Technol. 50, 702–734. doi: 10.1111/bjet.12688

[ref012] Ratelle C. F. Senécal C. Vallerand R. J. Provencher P. (2005). The relationship between school-leisure conflict and educational and mental health indexes: A motivational analysis. J. Appl. Soc. Psychol. 35, 1800–1822. doi: 10.1111/j.1559-1816.2005.tb02196.x

[ref010] Redmond P. Lock J. V. (2006). A flexible framework for online collaborative learning. Internet High. Educ. 9, 267–276. doi: 10.1016/j.iheduc.2006.08.003

[ref08] Reinders H. (2010). Towards a classroom pedagogy for learner autonomy: A framework of independent language learning skills. Aust. J. Teach. Educ 35, 40–55. doi: 10.14221/ajte.2010v35n5.4

[ref84] Reinders H. Darasawang P. (2012). “Diversity in language support” in Computer-assisted language learning: Diversity in research and practice. ed. Stockwell G. (Cambridge: Cambridge University Press), 49–70.

[ref85] Reinders H. White C. (2011). Learner autonomy and new learning environments. Lang. Learn. Technol. 15, 1–3. doi: 10125/44254

[ref86] Richardson J. T. E. (2006). Investigating the relationship between variations in students’ perceptions of their academic environment and variations in study behaviour in distance education. Br. J. Educ. Psychol. 76, 867–893. doi: 10.1348/000709905X69690, PMID: 17094890

[ref87] Roorda D. L. Koomen H. M. Spilt J. L. Oort F. J. (2011). The influence of affective teacher–student relationships on students’ school engagement and achievement a meta-analytic approach. Rev. Educ. Res. 81, 493–529. doi: 10.3102/0034654311421793

[ref88] Rubin K. H. Bukowski W. M. Bowker J. C. (2015). “Children in peer groups” in Handbook of child psychology and developmental science. ed. Lerner R. M., vol. 4 (Hoboken, NJ: Wiley), 1–48.

[ref89] Ryan R. M. Deci E. L. (2000). Self-determination theory and the facilitation of intrinsic motivation, social development, and well-being. Am. Psychol. 55, 68–78. doi: 10.1037/0003-066X.55.1.68, PMID: 11392867

[ref90] Sabah N. M. (2016). Exploring students’ awareness and perceptions: influencing factors and individual differences driving m-learning adoption. Comput. Hum. Behav. 65, 522–533. doi: 10.1016/j.chb.2016.09.009

[ref91] Saini M. K. Goel N. (2019). How smart are smart classrooms? A review of smart classroom technologies. ACM Comput. Surv. 52, 1–28. doi: 10.1145/3365757

[ref92] Schaufeli W. B. Martínez I. M. Pinto A. M. Salanova M. Bakker A. B. (2002). Burnout and engagement in university students: a cross-national study. J. Cross-Cult. Psychol. 33, 464–481. doi: 10.1177/0022022102033005003

[ref93] Schunk D. H. (1991). Self-efficacy and academic motivation. Educ. Psychol. 26, 207–231. doi: 10.1080/00461520.1991.9653133

[ref06] Shadiev R. Liu T. Hwang W.-Y. (2020). Review of research on mobile-assisted language learning in familiar, authentic environments. Br. J. Educ. Technol. 51, 709–720. doi: 10.1111/bjet.12839

[ref94] Sharan Y. (2015). Meaningful learning in the cooperative classroom. Education 43, 83–94. doi: 10.1080/03004279.2015.961723

[ref011] Skinner E. A. Pitzer J. R. (2012). “Developmental dynamics of student engagement, coping, and everyday resilience,” in Handbook of Research on Student Engagement. eds. Christenson S. L. Reschly A. L. Wylie C. (Boston, MA: Springer), 21–44.

[ref95] Tabachnick B. G. Fidell L. S. (2007). Using multivariate statistics. New York: Allyn and Bacon/Pearson Education.

[ref96] Teo T. (2010). A path analysis of pre-service teachers’ attitudes to computer use: applying and extending the technology acceptance model in an educational context. Interact. Learn. Environ. 18, 65–79. doi: 10.1080/10494820802231327

[ref97] Teo T. Noyes J. (2014). Explaining the intention to use technology among pre-service teachers: a multi-group analysis of the unified theory of acceptance and use of technology. Interact. Learn. Environ. 22, 51–66. doi: 10.1080/10494820.2011.641674

[ref98] Teo T. van Schaik P. (2012). Understanding the intention to use technology by preservice teachers: an empirical test of competing theoretical models. Int. J. Hum.-Com. Int. 28, 178–188. doi: 10.1080/10447318.2011.581892

[ref02] Thorburn M. Stolz S. A. (2021). Emphasising an embodied phenomenological sense of the self and the social in education. Br. J. Educ. Stud. 69, 365–380. doi: 10.1080/00071005.2020.1796923

[ref99] Usher E. L. Pajares F. (2009). Sources of self-efficacy in mathematics: a validation study. Contemp. Educ. Psychol. 34, 89–101. doi: 10.1016/j.cedpsych.2008.09.002

[ref100] Waheed H. Hassan S. U. Aljohani N. R. Hardman J. Alelyani S. Nawaz R. (2020). Predicting academic performance of students from VLE big data using deep learning models. Comput. Hum. Behav. 104:106189. doi: 10.1016/j.chb.2019.106189

[ref101] Xia W. Lee G. (2000). “The influence of persuasion, training and experience on user perceptions and acceptance of IT innovation,” in *Proceedings of the 21st International Conference on Information Systems, (Atlanta: Association for Information Systems)*. pp. 371–384.

[ref102] Xie K. Heddy B. C. Greene B. A. (2019). Affordances of using mobile technology to support experience-sampling method in examining college students’ engagement. Comput. Educ. 128, 183–198. doi: 10.1016/j.compedu.2018.09.020

[ref103] Xu Z. Z. Qi C. (2019). The relationship between teacher-student relationship and academic achievement: the mediating role of self-efficacy. Eur. J. Math. Sci. Tech. Educ. 15, 1–7. doi: 10.29333/ejmste/105610

[ref104] Yang C. (2018). A formalist perception on language acquisition. Ling. Approach. Biling. 8, 665–706. doi: 10.1075/lab.18014.yan

[ref105] Yeoman P. Wilson S. (2019). Designing for situated learning: understanding the relations between material properties, designed form and emergent learning activity. Br. J. Educ. Technol. 50, 2090–2108. doi: 10.1111/bjet.12856

[ref106] Yesilyurt E. Ulas A. H. Akan D. (2016). Teacher self-efficacy, academic self-efficacy, and computer self-efficacy as predictors of attitude toward applying computer-supported education. Comput. Hum. Behav. 64, 591–601. doi: 10.1016/j.chb.2016.07.038

[ref107] Yilmaz M. Gurcay D. Ekici G. (2007). Adaptation of the academic self-efficacy scale to Turkish. Hacet. Univ. J. Educ. 33, 253–259.

[ref07] Zhao H. Sullivan K. P. H. Mellenius I. (2014). Participation, interaction and social presence: An exploratory study of collaboration in online peer review groups. Br. J. Educ. Technol. 45, 807–819. doi: 10.1111/bjet.12094

[ref108] Zuffianò A. Alessandri G. Gerbino M. Kanacri B. P. L. Di Giunta L. Milioni M.. (2013). Academic achievement: the unique contribution of self-efficacy beliefs in self-regulated learning beyond intelligence, personality traits, and self-esteem. Learn. Individ. Differ. 23, 158–162. doi: 10.1016/j.lindif.2012.07.010

